# A new genus and species of leptonetid spiders (Araneae, Leptonetidae) from Guangdong Province, China

**DOI:** 10.3897/BDJ.10.e80219

**Published:** 2022-01-20

**Authors:** Kuiwen Yang, Hanchao Li, Yanfeng Tong, Dongju Bian

**Affiliations:** 1 College of Life Science, Shenyang Normal University, Shenyang 110034, China College of Life Science, Shenyang Normal University Shenyang 110034 China; 2 Key Laboratory of Forest Ecology and Management, Institute of Applied Ecology, Chinese Academy of Sciences, Shenyang 110016, China Key Laboratory of Forest Ecology and Management, Institute of Applied Ecology, Chinese Academy of Sciences Shenyang 110016 China

**Keywords:** biodiversity, new taxa, taxonomy, Asia

## Abstract

**Background:**

The spider family Leptonetidae Simon, 1890 includes 20 genera and 366 species from North America, the Mediterranean Region and Asia. Currently, 132 species belonging to six genera have been recorded in China.

**New information:**

A new genus and species of leptonetid spiders, *Yueleptonetadongxing* gen. et sp. n., is described from Guangdong Province, China. *Yueleptoneta* gen. n. is distinct from the other genera in the chelicerae having the stridulatory file on the lateral margin and the male palp having a tarsal spur, lacking strong spines or apophyses on the femur and tibia.

## Introduction

Members of the family Leptonetidae Simon, 1890 are tiny (1–3 mm) and typically have six eyes, with posterior median eyes displaced behind the anterior lateral eyes and posterior lateral eyes, anterior median eyes lost. Most species live in moist habitats, such as leaf litter, under rocks and especially in caves (Ledford et al. 2021).

Leptonetidae is represented in China by 132 species belonging to six genera: *Falcileptoneta* Komatsu, 1970 (9 spp.), *Jingneta* Wang & Li, 2020 (9 spp.), *Leptonetela* Kratochvíl, 1978 (105 spp.), *Longileptoneta* Seo, 2015 (6 spp.), *Masirana* Kishida, 1942 (1 sp.) and *Rhyssoleptoneta* Tong & Li, 2007 (2 spp.) (Wang et al. 2020, Zhu and Li 2021, WSC 2022).

In this paper, a new genus and species of leptonetid spiders, collected from the leaf litter in Guangdong Provnice of China, is described and illustrated.

## Materials and methods

The specimens were examined using a Leica M205C stereomicroscope. Details were studied under an Olympus BX51 compound microscope. Photomicroscope images were made with a Canon EOS 750D zoom digital camera (18 megapixels) mounted on an Olympus BX51 compound microscope. Photos were stacked with Helicon Focus 6.7.1 and processed in Adobe Photoshop CC 2020. For scanning electron microscopy (SEM), specimens were air-dried, sputter-coated using IXRF SYSTEMS and imaged with a Hitachi TM3030 SEM. Leg measurements are shown as: total length (femur, patella, tibia, metatarsus, tarsus) and, when missing, was coded as "–". Palp measurements are shown as: total length (femur, patella, tibia, tarsus). All measurements were taken using an Olympus BX51 compound microscope and are in millimetres.

All specimens are preserved in 75% ethanol. The type material is deposited in the College of Life Science, Shenyang Normal University (SYNU) in Liaoning, China.

The following abbreviations are used in the text and figures: AER = anterior eye row; ALE = anterior lateral eyes; ALE–PME = distance between ALE and PME; At = atrium; Co = conductor; Em = embolus; PER = posterior eye row; PLE = posterior lateral eyes; PLE–PLE = distance between PLE and PLE; PLE–PME = distance between PLE and PME; PME = posterior median eyes; Sd = sperm duct; Ser = serrula; Sp = spermathecae; Str = stridulatory file; Ts = tarsal spur.

## Taxon treatments

### Yueleptoneta

Tong gen. n.

670671CA-C5FC-5904-9D1B-E6FE80146529

48D97727-253F-49F3-B682-63811BDE1C9D

Yueleptoneta Yueleptoneta dongxing Yang, Tong & Bian Status: new species described in this paper.

#### Description

**Figure 1.**
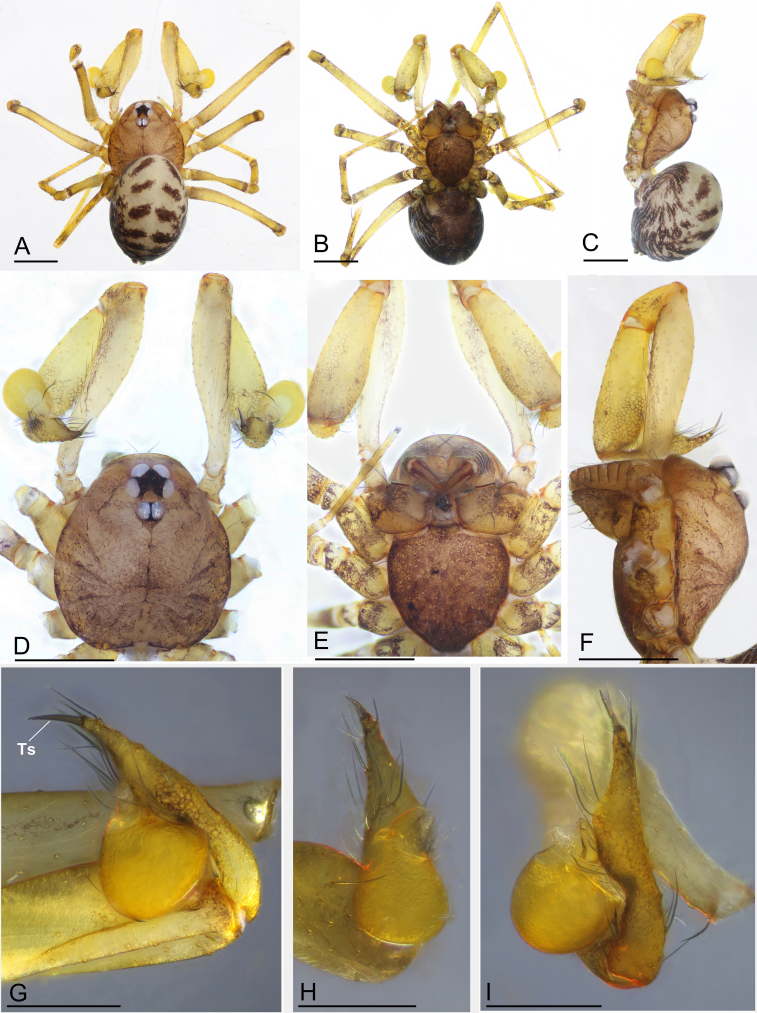
*Yueleptonetadongxing* sp. n., holotype male. **A** habitus, dorsal view; **B** habitus, ventral view; **C** habitus, lateral view; **D** prosoma, dorsal view; **E** prosoma, ventral view; **F** prosoma, lateral view; **G** left palp, retrolateral view; **H** left palp, ventral view; **I** left palp, dorsal view. Abbreviation: Ts = tarsal spur. Scale bars: 0.4 mm (A–F) and 0.2 mm (G–I).

**Figure 2.**
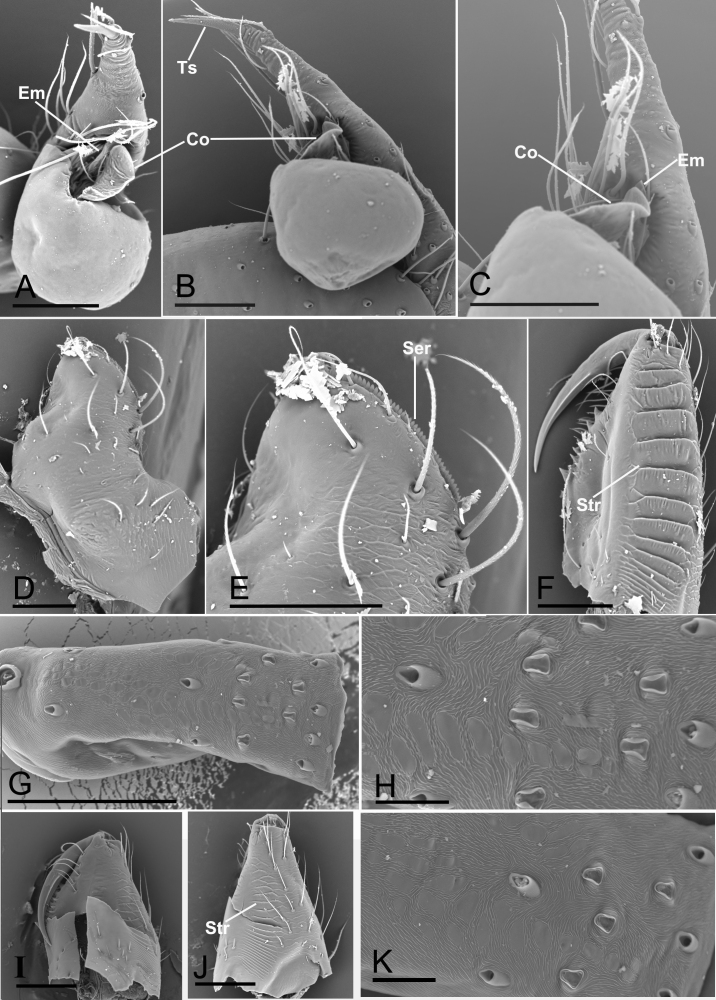
*Yueleptonetadongxing* sp. n., holotype male (A–H), paratype female (I–K), SEM. **A** left palp, ventral view; **B** left palp, retrolateral view; **C** detail of left palp, retrolateral view; **D** endite, ventral view; **E** detail of endite, ventral view; **F** left chelicera, posterior view; **G** patella III, dorsal view; **H** detail of patella III, dorsal view; **I** left chelicera, posterior view; **J** left chelicera, lateral view; **K** detail of patella III, dorsal view. Abbreviations: Co = conductor, Em = embolus, Ser = serrula, Str = stridulatory file, Ts = tarsal spur. Scale bars: 0.1 mm (A–G, I, J) and 0.02 mm (H, K).

**Figure 3.**
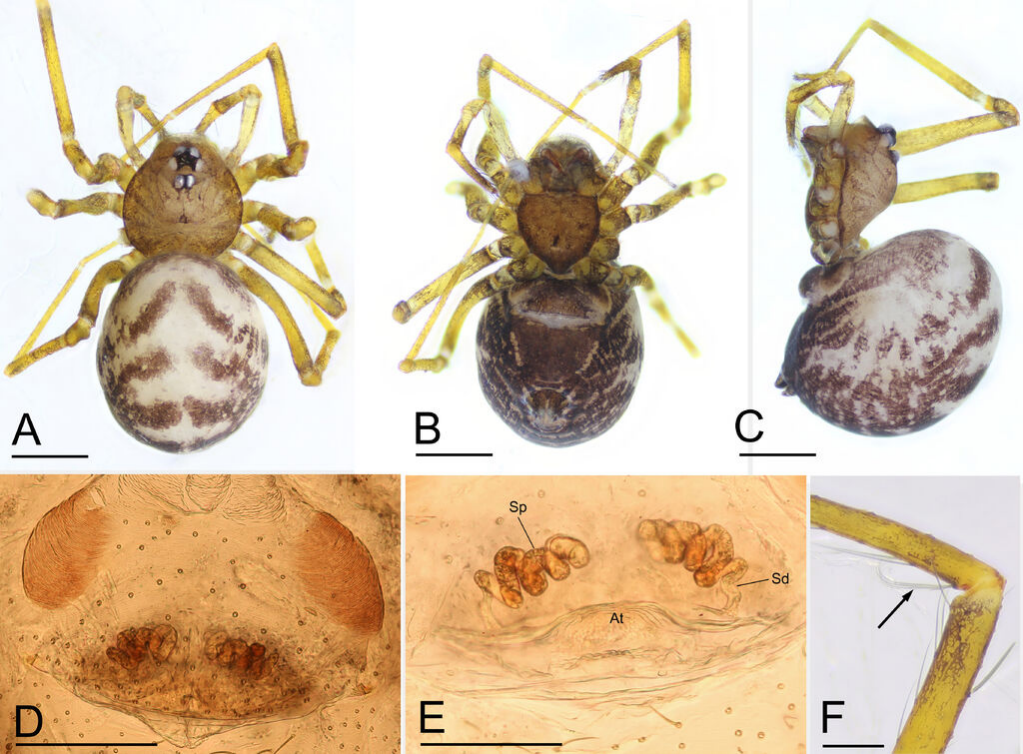
*Yueleptonetadongxing* sp. n., paratype female. **A** habitus, dorsal view; **B** habitus, ventral view; **C** habitus, lateral view; **D** genitalia, ventral view; **E** genitalia, dorsal view; **F** metatarsus II, lateral view, arrow shows preening comb. Abbreviations: At = atrium, Sd = sperm duct, Sp = spermatheca. Scale bars: 0.4 mm (A–C), 0.2 mm (D–E) and 0.1 mm (F).

Carapace brown and median groove needle-shaped, distinct. Six-eyed (Fig. 1D and Fig. 3A). ALE and PLE contiguous, PME posteriorly displaced. Chelicera with stridulatory file on the lateral margin (Fig. 2F, I and J). Endite anterior margin with serrula (Fig. 2D and E). Patellar gland oval (Fig. 2G, H and K). Opisthosoma with distinct patterns. Palp (Fig. 1G–I and Fig. 2A–C): femur and tibia without strong spines; tarsus with a spur at tip. Palpal bulb with a leaf-shaped embolus and a membranous, flattened conductor. Female internal genitalia with a pair of highly coiled spermathecae and sperm ducts (Fig. 3D and E).

#### Diagnosis

Male of the genus *Yueleptoneta* gen. n. is similar to *Leptonetela* Kratochvíl, 1978 and *Longileptoneta* Seo, 2015 in having a strong palpal tarsal spur (Fig. 1G–I, Fig. 2A and B), but can be distinguished from both genera by the palpal femur, tibia and tarsus lacking strong spines (Fig. 1D–I) (vs. palpal tibia with a row of spines in *Leptonetela* (Wang et al. 2017: figs 4C and D) and palpal femur with strong spines and palpal tarsus with a prolateral distal spur in *Longileptoneta* (Seo 2015: figs 1C–G)) and the chelicerae having the stridulatory file on the lateral margin (Fig. 2F) (vs. lacking stridulatory file in both genera). Male of the new genus is also similar to *Leptoneta* Simon, 1872 in the palpal femur and tibia lacking strong spines, but can be distinguished by having the palpal tarsal spur (vs. lacking palpal tarsal spur, but having strip-shaped appendices in *Leptoneta* (Le Peru 2011: fig. 118)). Female of the new genus is similar to *Leptonetela* Kratochvíl, 1978 by having strongly coiled spermathecae (Fig. 3E), but can be distinguished by the chelicerae having the stridulatory file on the lateral margin (Fig. 2I and J).

#### Etymology

The generic name is derived from the Pinyin word “Yue”, referring to Guangdong Province (Yue is a short name for Guangdong), where the material has been collected and the genus name *Leptoneta* Simon. The gender is feminine.

#### Distribution

China (Guangdong).

### Yueleptoneta dongxing

Yang, Tong & Bian sp. n.

DACD4A80-4151-5CA4-958A-A20CD121A0AB

1149790B-DC13-4697-AF09-15E761E573E8

#### Materials

- **Type status:** Holotype. **Occurrence:** individualCount: 1; sex: male; lifeStage: adult; **Taxon:** scientificName: *Yueleptonetadongxing*; order: Araneae; family: Leptonetidae; genus: Yueleptoneta; scientificNameAuthorship: Yang, Tong & Bian; **Location:** country: China; stateProvince: Guangdong; county: Heyuan City; locality: Xinhuilong Town, Dongxing Village, Wanlvgu Resort Area; verbatimElevation: 160 m a.s.l.; verbatimCoordinates: 23°42'44"N, 114°38'5"E; **Event:** samplingProtocol: sifting leaf litter; eventDate: 24 April 2021
- **Type status:** Paratype. **Occurrence:** individualCount: 4; sex: female; lifeStage: adult; **Taxon:** scientificName: *Yueleptonetadongxing*; order: Araneae; family: Leptonetidae; genus: Yueleptoneta; scientificNameAuthorship: Yang, Tong & Bian; **Location:** country: China; stateProvince: Guangdong; county: Qingyuan City, Yingde City; locality: Donghua Temple; verbatimElevation: 157 m a.s.l.; verbatimCoordinates: 24°9'15"N, 113°26'36"E; **Event:** samplingProtocol: sifting leaf litter; eventDate: 5 April 2021

#### Description

Male. Total length 1.81 (Fig. 1A–C). Carapace 0.83 long, 0.71 wide. Opisthosoma 0.96 long, 0.75 wide. Prosoma brown (Fig. 1D–F). Median groove dark brown, needle-shaped. Cervical grooves and radial furrows distinct. Clypeus 0.15 high, slightly sloped anteriorly. Six-eyed (Fig. 1D), ALE and PLE connected to each other by the black bases, PME separated from ALE and PLE. Eye sizes: ALE 0.09, PLE 0.08, PME 0.07. Distance between eyes: ALE–PME 0.09, PLE–PLE 0.09, PLE–PME 0.03. AER 0.15, PER 0.19. Chelicerae light brown, with stridulatory file on lateral margin; fang groove with 1 large and 7 small teeth at promargin and 4 small teeth on retromargin (Fig. 2F). Endites light brown, anterior margin with serrula (Fig. 2D and E). Labium dark brown, fused to sternum. Sternum oval, brown. Legs yellowish. Leg measurements: I - (1.14, 0.24, -, -, -); II - (0.93, 0.23, -, -, -); III - (0.78, 0.22, -, -, -); IV 3.78 (1.01, 0.23, 1.01, 0.95, 0.58). Opisthosoma pale yellow, ovoid, with distinct brown patterns. Palp (Fig. 1G–I and Fig. 2A–C). Measurements: 2.15 (0.86, 0.28, 0.55, 0.46). Femur without any spines; tibia without apophysis; tarsus with a spur at tip; palpal bulb oval in shape, smooth; conductor membranous, flattened; embolus leaf-shaped.

Female. Similar to male in general features (Fig. 2I–K and Fig. 3A–C). Total length 1.62. Carapace 0.66 long, 0.62 wide. Opisthosoma 1.02 long, 0.87 wide. Eye sizes: ALE 0.08, PLE 0.07, PME 0.06. Distance between eyes: ALE–PME 0.09, PLE–PLE 0.09, PLE–PME 0.03. AER 0.14, PER 0.18. Clypeus 0.10 high. Leg measurements: I 2.78 (0.73, 0.21, 0.74, 0.63, 0.47); II 2.35 (0.63, 0.21, 0.54, 0.58, 0.39); III 2.05 (0.59, 0.20, 0.45, 0.47, 0.34); IV 2.74 (0.75, 0.21, 0.71, 0.65, 0.42). Leg formula: I–IV–II–III. Metatarsus II with ventro-apical preening comb (Fig. 3F). Internal genitalia consisting of paired spermathecae and sperm ducts. Spermathecae highly coiled (Fig. 3D and E).

#### Diagnosis

Specimens of *Yueleptonetadongxing* sp. n. are distinguished by the characters discussed in the genus diagnosis section.

#### Etymology

The specific name is a noun in apposition and refers to the type locality.

## Supplementary Material

XML Treatment for Yueleptoneta

XML Treatment for Yueleptoneta dongxing
